# Genome-Wide Identification of the ERF Transcription Factor Family for Structure Analysis, Expression Pattern, and Response to Drought Stress in *Populus alba × Populus glandulosa*

**DOI:** 10.3390/ijms24043697

**Published:** 2023-02-12

**Authors:** Dong Zeng, Li-Juan Dai, Xiang Li, Wei Li, Guan-Zheng Qu, Shuang Li

**Affiliations:** 1 State Key Laboratory of Tree Genetics and Breeding, Northeast Forestry University, Harbin 150040, China; 2 School of Agriculture, Ningxia University, Yinchuan 750021, China

**Keywords:** *Populus alba × Populus glandulosa*, drought stress, transcriptomic sequencing, ERF family, expression pattern

## Abstract

The Ethylene Responsive Factor (ERF) transcription factor family is important for regulating plant growth and stress responses. Although the expression patterns of ERF family members have been reported in many plant species, their role in *Populus alba × Populus glandulosa*, an important model plant for forest research, remains unclear. In this study, we identified 209 PagERF transcription factors by analyzing the *P. alba × P. glandulosa* genome. We analyzed their amino acid sequences, molecular weight, theoretical pI (Isoelectric point), instability index, aliphatic index, grand average of hydropathicity, and subcellular localization. Most PagERFs were predicted to localize in the nucleus, with only a few PagERFs localized in the cytoplasm and nucleus. Phylogenetic analysis divided the PagERF proteins into ten groups, Class I to X, with those belonging to the same group containing similar motifs. Cis-acting elements associated with plant hormones, abiotic stress responses, and MYB binding sites were analyzed in the promoters of *PagERF* genes. We used transcriptome data to analyze the expression patterns of *PagERF* genes in different tissues of *P. alba × P. glandulosa*, including axillary buds, young leaves, functional leaves, cambium, xylem, and roots, and the results indicated that *PagERF* genes are expressed in all tissues of *P. alba × P. glandulosa*, especially in roots. Quantitative verification results were consistent with transcriptome data. When *P. alba × P. glandulosa* seedlings were treated with 6% polyethylene glycol 6000 (PEG6000), the results of RT-qRCR showed that nine *PagERF* genes responded to drought stress in various tissues. This study provides a new perspective on the roles of PagERF family members in regulating plant growth and development, and responses to stress in *P. alba × P. glandulosa*. Our study provides a theoretical basis for ERF family research in the future.

## 1. Introduction

Transcription factors are located in the nucleus and play important roles in regulating plant growth, development and abiotic stress response [1]. They are divided into families according to their specific conserved protein domains, such as Apetala2/ethylene responsive factor (AP2/ERF), myeloblastosis (MYB), [NAM (No apical meristem)/ATAF1/2 (Arabidopsis transcription activation factor 1/2)/CUC2 (cup-shaped cotyledon 2)] (NAC), basic region/leucine zipper (bZIP), etc. [2]. AP2/ERF is the largest transcription factor superfamily in plants and is found in many species, such as *Bryum argenteum* [3], *Fagopyum tataricum* [4], *Prunus mume* [5], and *Phyllostachys edulis* [6]. The AP2/ERF superfamily is divided into Apetala2 (AP2), Ethylene Responsive Factor (ERF), Related to Abscisic Acid Insensitive 3/Viviparous 1 (RAV), and soloist families according to the number and sequence similarity of AP2 domains [7]. The AP2 family contains two conserved AP2 domains, and its regulatory role in flower [8] and seed [9] development is widely reported. ERF family members contain a conserved AP2 domain and are involved in the regulation of plant growth, development, and response to abiotic stress [10]. The ERF family is divided into ERF and Dehydration-Responsive Element Binding (DREB) subfamilies according to cis-acting elements associated with ERF binding; ERF subfamily members bind to the ethylene response element GCC-box, while DREB subfamily members bind to the drought and low temperature response element DRE [11]. The RAV family is characterized by a conserved AP2/ERF domain at the N-terminus and a B3 domain at the C-terminus [12], while the soloist family contains an AP2/ERF conserved domain, but its gene structure is significantly different from that of other subfamilies [13]. AP2/ERF family members are important transcription factors in plants, and their functions cannot be replaced.

The ERF family is the most abundant family in the AP2/ERF superfamily and is widely involved in plant responses to drought stress [14]; members of this family have been identified in *Hordeum vulgare* L. [15], *Zea mays* L. [16], and *Raphanus sativus* L. [17]. Studies have shown that ERF transcription factors respond to drought stress by participating in the signal transduction pathways of plant hormones, including cytokinins [18], jasmonates [19], and ethylene [20]. For example, TINY is a transcription factor in the ERF family in *Arabidopsis thaliana*. TINY is negatively regulated by brassinosteroid signaling through BIN2 phosphorylation and responds to drought stress positively [21]. ERF transcription factors are also involved in the MAPK signaling pathway regulating plant responses to drought stress [22]. When plants are subjected to drought stress, various types of protein kinase regulate the steady-state environment and coordinate changes in reactive oxygen species to respond to drought stress by regulating the transcription factors of multiple families, including ERF [23]. OsERF48 interacts with calcium-binding protein and other protein kinases in the CDPK signaling pathway to respond to drought [24]. ERF transcription factors are also involved in regulating the biosynthesis of secondary metabolites in response to drought [25]. For example, MdERF38 interacts with MdMYB1 to promote anthocyanin biosynthesis in *Malus* × *domestic* under drought stress [26]. Overall, the ERF family is an important transcription factor family in drought stress response regulation.

Due to its characteristics of fast growth and high drought and salt tolerance, the poplar (*Populus* spp.) is widely used as an important raw material for papermaking and other production activities [27]. *Populus alba × Populus glandulosa* is a fast-growing hybrid poplar widely distributed in Korea and China [28]. In recent years, *P. alba × P. glandulosa* has been used extensively as a wood material for the study of plant growth and gene function owing to its high genetic transformation rate [29,30]. Moreover, the emergence of high-quality genomes of *P. alba × P. glandulosa* [31,32] revealed that *P. alba × P. glandulosa* has 19 pairs of chromosomes divided into two subgenomes, A (from *P. alba*) and B (from *P. glandulosa*). These studies provide a theoretical basis for further use of *P. alba × P. glandulosa* as a model tree and lay the foundation for molecular research of trees.

The ERF family is the largest family in the AP2/ERF superfamily and has been widely reported in *Manihot esculenta* Crantz [33] and *Ipomoea batatas* (L.) Lam [34]; for example, 170 ERFs have been identified in *P. trichocarpa* [35] and 147 ERFs have been identified in *Manihot esculenta* [36]. Although ERF transcription factors in *Populus trichocarpa* were identified [37] and shown to respond to abiotic stresses such as salt stress [11], the molecular mechanism of ERF transcription factors in response to drought stress in poplar remains unknown. Furthermore, the fast-growing and efficient genetic transformation characteristics of *P. alba × P. glandulosa* make it a model plant for forest research. Consequently, it is critical to investigate the drought resistance function and molecular mechanism of ERF family transcription factors in *P. alba × P. glandulosa*. In this study, we systematically identified members of the ERF family in *P. alba × P. glandulosa*, analyzed their physicochemical properties, evolutionary relationships, chromosome location, and gene structure. In addition, we used transcriptome data from axillary buds, young leaves, functional leaves, cambium, xylem, and roots of *P. alba × P. glandulosa* to analyze the tissue specificity of *PagERF* genes and used 6% PEG6000 to simulate drought stress. This study therefore provides a theoretical basis for future research on the mechanisms of PagERF regulation of plant growth and development and responses to drought in *P. alba × P. glandulosa*.

## 2. Results

### 2.1. Identification of the PagERF Transcription Factor Family in P. alba × P. glandulosa

**Figure 1:**
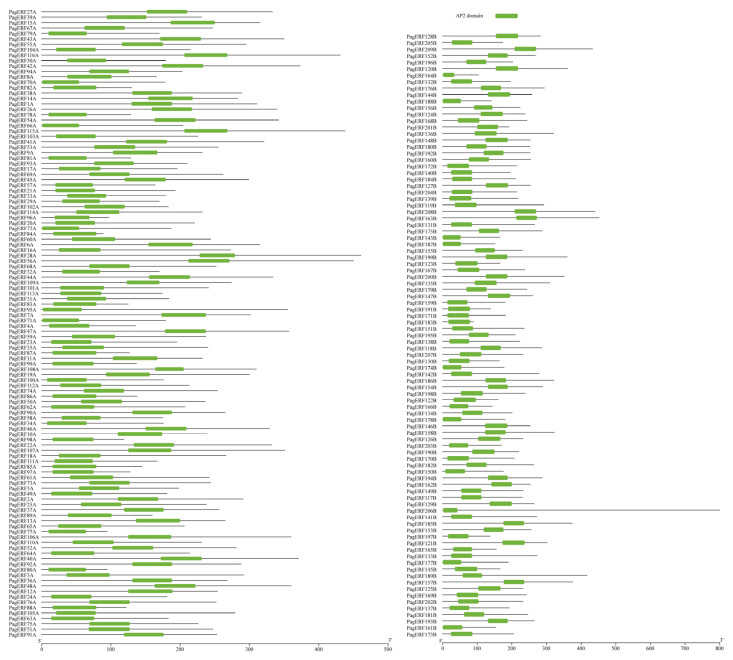
Physiological analysis of the conserved domain of PagERF transcription factors. Green rectangles indicate the AP2 domain. The X-axis indicates the length of protein sequence.

To identify members of the PagERF family of transcription factors in *P. alba × P. glandulosa*, we compared protein sequences from *P. alba × P. glandulosa* with sequences representing AtAP2/ERF superfamily proteins in *Arabidopsis thaliana*; protein sequences with sequence identity (percentage identity) greater than 80% were considered as candidate sequences. According to the characteristics of ERF family protein sequences, we identified 209 PagERF proteins containing only one AP2 domain through conserved domain analysis (Figure 1), named PagERF1A to PagERF209B. We analyzed the following molecular properties of these protein sequences: number of amino acids, molecular weight, theoretical pI, instability index, aliphatic index, grand average of hydropathicity, and subcellular localization (Supplementary Table S1). The longest protein, PagERF206B, comprised 762 amino acids and its subcellular localization was in the nucleus. The shortest proteins, PagERF84A (molecular weight: 10,264.9 kDa) and PagERF183B (molecular weight: 10,278.93 kDa), each comprised 89 amino acids and by use of the Plant-mPLoc tool were found to be located in both the cytoplasm and the nucleus (Supplementary Table S1).

### 2.2. Chromosomal Distribution of PagERF Genes

**Figure 2:**
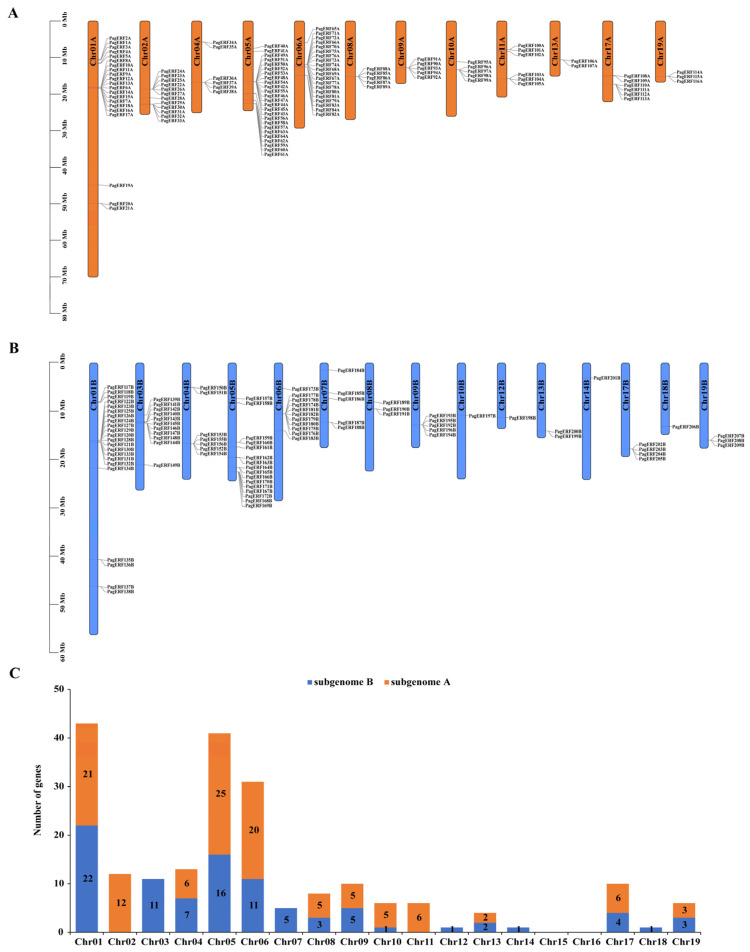
Chromosomal distribution of *PagERF* genes on chromosomes. (**A**,**B**) Distribution of *PagERF* genes among chromosomes of subgenome A (**A**) and subgenome B (**B**). (**C**) Distribution of *PagERF* genes among all chromosomes. Orange, subgenome A; blue, subgenome B.

The hybrid poplar *P. alba × P. glandulosa* possesses two subgenomes of 19 chromosomes, subgenome A and subgenome B. Identifying the distribution of *PagERF* genes on chromosomes of *P. alba × P. glandulosa* (Supplementary Table S2) revealed that *PagERF* genes are distributed across 17 chromosomes: 12 chromosomes in subgenome A (Figure 2A) and 15 chromosomes in subgenome B (Figure 2B), with no genes on chromosomes 15 or 16 in either subgenome. There were 116 *PagERF* genes in subgenome A and 93 *PagERF* genes in subgenome B (Figure 2C, Supplementary Figure S1). The most *PagERF* genes (43) were distributed on chromosome 1, 21 in subgenome A and 22 *PagERF* in subgenome B, followed by 41 *PagERF* genes on chromosome 5, 25 in subgenome A and 16 in subgenome B. *PagERF* genes on chromosome 2 and chromosome 11 belonged only to subgenome A; *PagERF* genes on chromosomes 3, 7, 12, 14, and 18 belonged only to subgenome B.

### 2.3. Phylogenetic Analysis of PagERF Proteins

**Figure 3:**
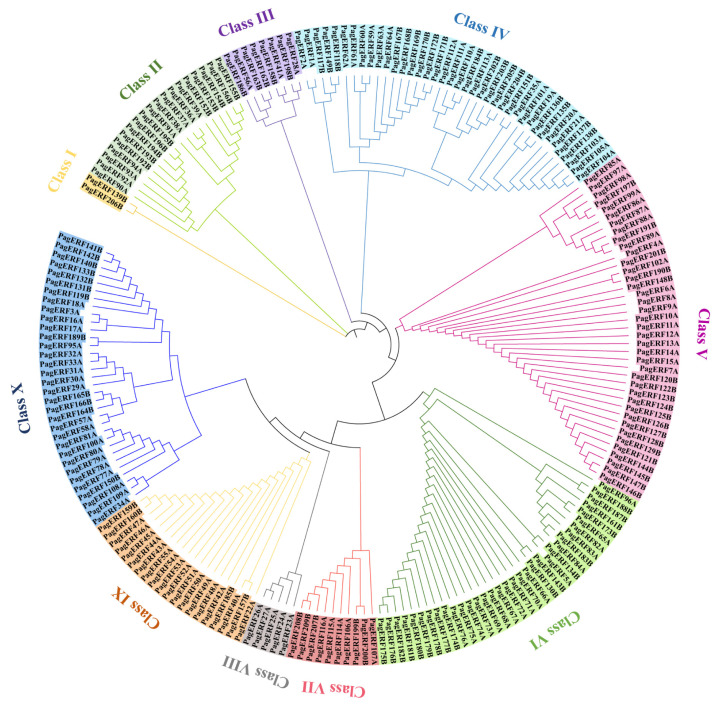
Phylogenetic tree of PagERF transcription factors. Different colors indicate subgroups of the PagERF family from Class I to X. The phylogenetic tree was built with MEGA 7.0 software (neighbor-joining algorithm, 1000 bootstrap replications).

To analyze the phylogenetic relationships of PagERF transcription factors, we reconstructed the phylogenetic tree of the PagERF proteins identified (Supplementary Table S3). We then divided the PagERF family into 10 subgroups (Figure 3), Classes I to X, according to their evolutionary relationships. Class I contained the fewest proteins, PagERF139B and PagERF206B, which belong to subgenome B; Class VIII contained five PagERF proteins, PagERF23A, PagERF24A, PagERF25A, PagERF26A, and PagERF27A, all belonging to subgenome A. Members of the other subgroups were associated with subgenomes A and B; Class IV and Class V were the largest subgroups, containing 39 members, followed by Class VI and Class X, containing 34 and 33 members, respectively.

### 2.4. Localization and Duplication of PagERF Genes

**Figure 4:**
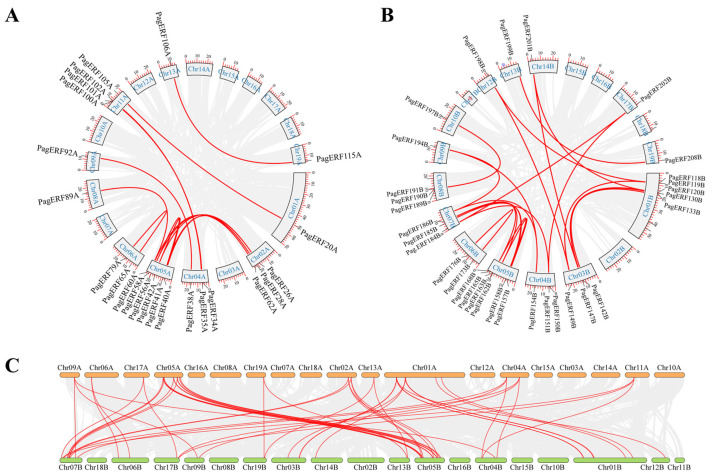
Gene localization and gene duplication of *PagERF* genes on chromosomes. (**A–C**) Localization and duplication of *PagERF* genes on chromosomes of subgenome A (**A**), subgenome B (**B**), and between subgenomes A and B (**C**). Grey lines indicate syntenic blocks between two regions of the *P. alba × P. glandulosa* subgenomes; red lines indicate relationships between *PagERF* genes.

To further explore the relationships among *PagERF* genes, we performed collinearity analysis on the replication events within the PagERF family. Eighteen gene pairs distributed on chromosome 2A and chromosome 5A showed the strongest relationships in subgenome A, while most of the other chromosomes had only one gene pair (Figure 4A). We also identified 25 gene pairs distributed on chromosome 1B and chromosome 3B, chromosome 4B and chromosome 7B, and chromosome 5B and chromosome 7B in subgenome B (Figure 4B). There were 38 gene pairs showing close relationships between chromosomes of subgenome A and subgenome B (Figure 4C), with *PagERF* genes displaying strong similarity on chromosomes 5A and 5B between the two subgenomes. According to the Ka and Ks value, the ratio of Ka/Ks between gene pairs was less than one except for *PagERF3A* and *PagERF119B* (Supplementary Tables S4–S6). In summary, the duplication events affecting *PagERF* genes in *P. alba × P. glandulosa* are complex, including events on chromosomes within subgenome A, within subgenome B, and between subgenome A and subgenome B.

### 2.5. Analysis of PagERF Gene Structure and PagERF Motif Composition

**Figure 5:**
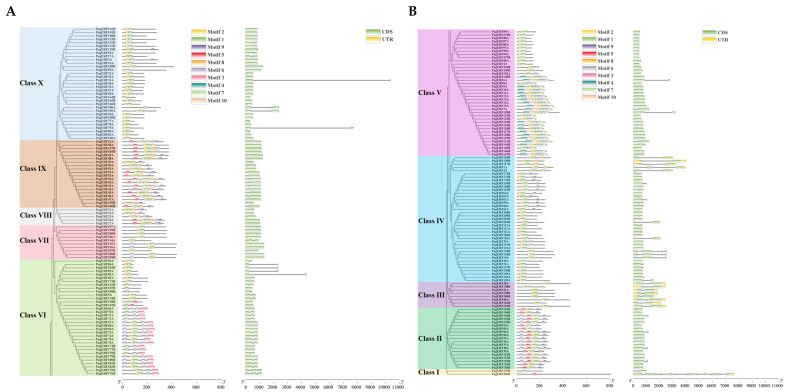
Motif and structure analysis of *PagERF* genes. (**A**,**B**) Protein motif and gene structure analysis of *PagERF* genes belonging to phylogenetic tree of Class VI~X (**A**) and Class I~V (**B**). Different colored boxes represent different motifs.

To further analyze the function of the PagERF family, we used the MEME tool to analyze the structure of *PagERF* genes and the protein motifs of the transcription factors (Figure 5). The E-value of a motif is based on its log likelihood ratio, width, sites, background letter frequencies, and the size of the training set. The gene structure of *PagERF* genes showed very complex characteristics; *PagERF31A* had the longest intron, *PagERF206B* contained the largest number of introns, and most *PagERF* genes contained no introns. PagERF proteins in the same subgroup in the phylogenetic tree had similar motifs, which we named motif 1 to motif 10 according to their E values from low to high, and the protein family membership of all ten motifs was analyzed using the InterPro tool and found to be ERF factor, of which motif 1 and motif 2 both enriched the ethylene-activated signaling pathway (GO:0009873) (Supplementary Table S7). We obtained the following findings: (I) The PagERF proteins containing motif 4 belonged to Class V, the PagERF proteins containing motif 3 belong to Class VI, and most of the Class IX PagERF proteins contained motifs 5, 2, 1, 8, and 6. (II) Most of the PagERFs in Class II contained motifs 6, 5, 2, 1, 9. We identified 10 motifs among all PagERF protein sequences, with each gene encoding from one to six motifs. (III) PagERF108A contained one motif, and PagERF22A and most PagERF proteins in Class V each contained six motifs.

### 2.6. Analysis of Cis-Acting Elements in PagERF Promoters

**Figure 6:**
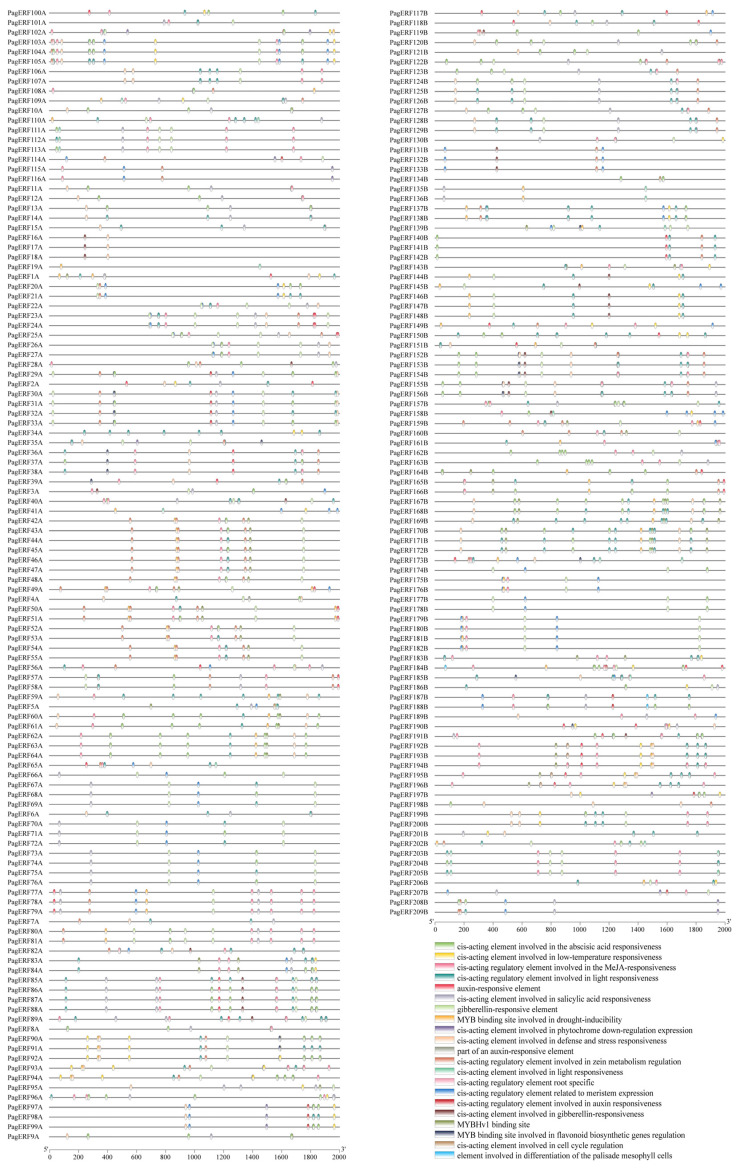
Analysis of cis-acting elements in *PagERF* promoters. Different colored boxes represent different cis-acting elements.

We used PlantCARE to analyze the *PagERF* promoters (Supplementary Table S9). Functional annotation revealed a total of 21 functional elements (Figure 6). Response elements for hormones including gibberellic acid, methyl jasmonate, and abscisic acid and auxin cis-acting elements, such as ABRE, ERE, of which there were eleven ABRE in the *PagERF150B* promoter. In addition, the promoters also contained a variety of cis-acting elements that respond to abiotic stresses including drought, salt, and low temperature, including MYB binding sites involved in drought-inducibility, such as ARE, DRE, myc. Surprisingly, most *PagERF* promoters had myc and the promoter of *PagERF16A*, *PagERF17A*, *PagERF18A*, *PagERF20A*, *PagERF21A*, and *PagERF131B*, *PagERF132B*, *PagERF133B*, *PagERF137B*, *PagERF138B* had ten myc in all (Supplementary Table S8). We also found cis-acting regulatory elements that were root specific, providing a research direction and theoretical basis for us to further explore the expression patterns of *PagERF* genes in different tissues of *P. alba × P. glandulosa* and their functions in response to abiotic stress.

### 2.7. Expression Pattern Analysis of PagERF Genes

**Figure 7:**
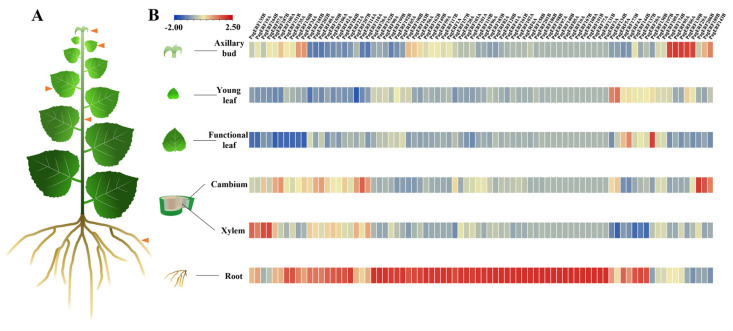
Expression patterns of *PagERF* genes in axillary buds, young leaves, functional leaves, cambium, xylem, and roots. (**A**) Schematic drawing for tissue sampling of *P. alba × P. glandulosa*. (**B**) Heatmap illustrating transcript patterns of *PagERF* genes from the transcriptomes of different tissues. Color scale from blue to red indicates expression level from low to high.

To explore the function of PagERF transcription factors in *P. alba × P. glandulosa*, we used RNA-seq data to analyze the expression patterns of *PagERF* genes in six different tissues: axillary bud, young leaf, functional leaf, cambium, xylem, and root (Figure 7A). *PagERF* genes had specific expression patterns in different tissues (Figure 7B, Supplementary Table S10). For example, most *PagERF* genes were strongly expressed in roots and weakly expressed in young leaves. In addition, *PagERF174B*, *PagERF95A*, *PagERF66A*, *PagERF139B*, and *PagERF29A* were strongly expressed in axillary buds, and *PagERF130B* was strongly expressed in the functional leaf. *PagERF206B* and *PagERF189B* were highly expressed in cambium, and *PagERF135B*, *PagERF164B*, and *PagERF57A* were highly expressed in xylem. These results indicated that *PagERF* genes are expressed in all tissues of *P. alba × P. glandulosa*, especially in roots.

### 2.8. RT-qPCR Validation of PagERF Gene Expression Patterns

**Figure 8:**
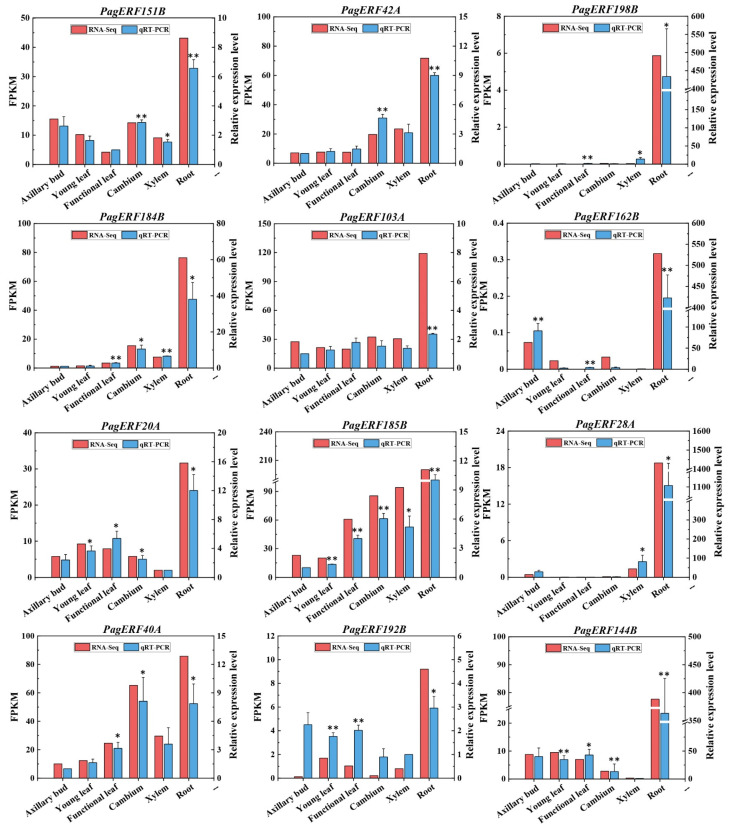
Quantitative validation of expression patterns for 12 *PagERF* genes in different tissues. The X-axis represents the different tissues. The left Y-axis indicates the FPKM value obtained by RNA-seq, and the right Y-axis indicates relative gene expression levels analyzed by RT-qPCR. Bars indicate mean ± SE (n = 3) from three independent trials. * *p* < 0.05, ** *p* < 0.01.

To verify the accuracy of transcriptome data, we randomly selected 12 *PagERF* genes for quantitative validation (Figure 8). The quantitative results were consistent with the RNA-seq results. Consequently, most of the genes were more strongly expressed in roots than other tissues, especially *PagERF198B* and *PagERF28A*. *PagERF40A* also showed strong expression in cambium, and *PagERF162B* had relatively strong expression in axillary buds. In addition, *PagERF42A*, *PagERF103A*, *PagERF151B*, and *PagERF185B* were substantially expressed in all tissues sampled. This indicated that *PagERF* genes have specific expression patterns in tissues of *P. alba × P. glandulosa*, especially in roots, which provided a basis for us to explore the expression patterns of *PagERF* genes in roots in response to drought.

### 2.9. Analysis of PagERF Genes in Response to Drought in Seedlings

**Figure 9:**
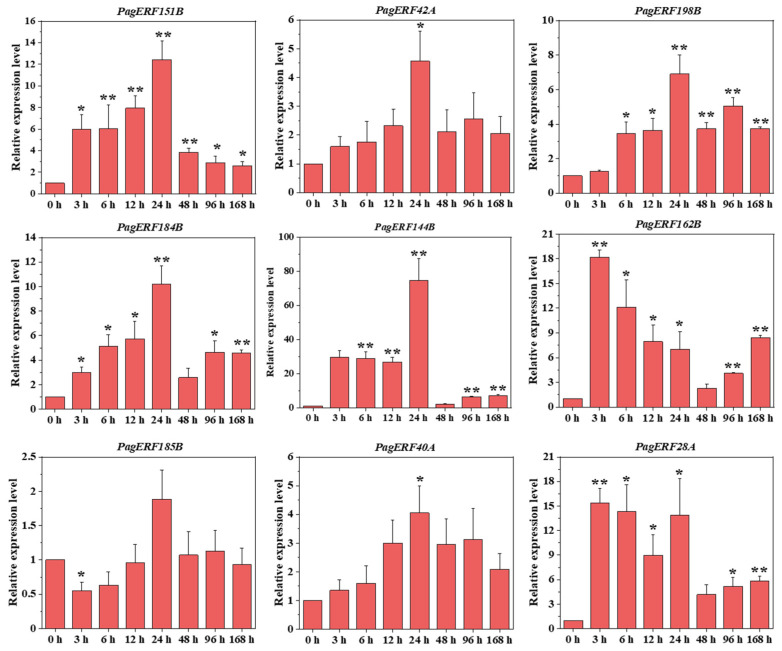
Quantitative validation of expression patterns for nine *PagERF* genes in roots under drought stress at eight time points. The X-axis represents samples under drought stress at eight time points. The Y-axis on the left indicates relative gene expression levels analyzed by RT-qPCR. Bars indicate mean ± SE (n = 3) from three independent trials. * *p* < 0.05, ** *p* < 0.01.

We used 6% PEG6000 treatment to simulate drought stress in *P. alba × P. glandulosa* and explored the response of nine *PagERF* genes in five tissues (axillary bud, young leaf, functional leaf, stem, and root) at eight time points using RT-qPCR (Figure 9, Supplementary Figures S2–S5). Most *PagERF* genes responded to drought in the roots but showed specific expression patterns at different time points. For example, expression of *PagERF162B* and *PagERF28A* was substantially up-regulated, and relative expression levels reached a maximum after 3 h under 6% PEG6000 treatment; meanwhile, *PagERF144B* showed relatively up-regulated expression levels at 24 h. However, some *PagERF* genes showed a down-regulated expression trend after drought treatment; for example, *PagERF185B* showed down-regulated expression within 12 h.

## 3. Discussion

AP2/ERF is a transcription factor superfamily that plays an essential role in plant growth and development in response to abiotic stress, and the function of ERF transcription factors in *P. alba × P. glandulosa* has not been studied, especially their regulatory roles in plant growth, development and responses to drought stress.

In this study, we identified 209 ERF transcription factors in *P. alba × P. glandulosa* according to their AP2 domain characteristics (Figure 1). Through phylogenetic analysis (Figure 3) and gene structure analysis (Figure 5), we determined that *PagERF* genes in the same phylogenetic group with similar motif distribution had widely varying length and distribution of coding sequences. We used the two subgenomes of *P. alba × P. glandulosa* to analyze the chromosomal localization of *PagERF* transcription factor genes (Figure 2, Supplementary Figure S1), which revealed complex intra-group and inter-group duplication relationships. In addition, we obtained eighteen gene pairs in subgenome A, 25 gene pairs in subgenome B, and 38 gene pairs between subgenome A and B using synteny analysis (Figure 4), and the ratio of Ka/Ks indicated that *P. alba × P. glandulosa* might undergo multiple selecting evolutionary directions and the gene pair of *PagERF3A* and *PagERF119B* might play a crucial role in the evolution (Supplementary Tables S4–S6). These results indicate that PagERF transcription factors may have similar functions in transcriptional regulation and interact closely. Owing to the particular characteristics of the *P. alba × P. glandulosa* genome [38], we speculate that the regulatory relationships among members of the PagERF family and other transcription factor families, such as MYB, in *P. alba × P. glandulosa* may be more complex than those in other poplars, including the molecular networks regulating plant growth and development or responding to abiotic stresses.

ERF transcription factors play an important role in regulating plant growth and development, including the biosynthesis of secondary metabolites [39,40,41] and the biosynthesis and transduction of plant hormones [42], and mutual regulation with microRNA [43]. For example, chrysanthemum (*Chrysanthemum morifolium*) CmERF053 regulates the germination of shoot branches by regulating auxin and cytokinin transport in the axillary bud [18] and Arabidopsis AtERF6 represses leaf growth by inhibiting cell division and cell expansion in the leaf [44]. ERF transcription factors also modify stem growth and wood properties [35] and change stem elongation and secondary xylem lignification [45]. In addition, ERF transcription factors such as rice (*Oryza sativa*) OsERF71 influence root growth by changing root structure, causing formation of enlarged aerenchyma, and regulating genes related to cell wall thickening and lignin biosynthesis [46]. In this study, we found that most PagERF promoters contain CAT-box, O2 site, which was associated with plant growth and development; ERE, AuxRR, and the TCA element were cis-elements associated with phytohormone responsiveness (Figure 6). This showed that *PagERF* genes may be regulated to play a critical role in plant growth and development. Also, we analyzed the expression patterns of *PagERF* genes in different tissues of *P. alba × P. glandulosa* (Figure 7B and Figure 8). This showed that most *PagERF* genes have high expression levels in roots, and a few genes are expressed in axillary bud, leaves, cambium, or xylem. Their expression patterns are relatively simple, being specifically expressed in certain tissues. For example, *PagERF174B*, *PagERF95A*, *PagERF66A*, *PagERF139B*, and *PagERF29A* show high expression levels in axillary buds; *PagERF5A* only displays high expression levels in young leaves; *PagERF130B* only shows high expression levels in functional leaves; *PagERF206B* and *PagERFF189B* have high expression levels only in cambium; *PagERF135B*, *PagERF19A*, *PagERF164B*, and *PagERF57A* display high expression levels only in xylem. Interestingly, regulation by ERF transcription factors has been reported in various plant tissues. Therefore, we hypothesize that PagERF transcription factors may specifically participate in the regulation of plant growth and development directly or indirectly in different tissues, especially roots, including plant hormone regulation and transcriptional regulation of binding to other transcription factors.

ERF transcription factors also play important regulatory roles in plant responses to abiotic stresses [47], including drought [48,49], cold [50,51], and salt [52,53]. In this study, we found that most PagERF promoters contain DRE elements, ABRE elements, and other elements involved in the response to stress (Figure 6); some PagERF promoters also contained MYB binding sites associated with the response to drought. We used PEG treatment to simulate drought stress conditions for different periods and examined expression of nine *PagERF* genes in various tissues of *P. alba × P. glandulosa* in response to drought using RT-qPCR (Figure 9, Supplementary Figures S2–S5). *PagERF* genes showed a strong response to drought in all tissues, especially roots. We speculate that the great changes in expression patterns of *PagERF* genes in response to abiotic stress may result from abnormal changes in plant hormone signals [54], such as gibberellins, cytokinins, and brassinosteroids, as well as MAPK signal transduction [55]. Moreover, homeostasis of the molecular network of transcriptional regulation in plants may be challenged under abiotic stress, affecting the transcriptional regulation process associated with PagERF proteins, further causing a series of defensive responses in various plant parts or organs. For example, differentiation of cambium cells and the growth process of xylem may be disturbed [56]; the role of PagERF proteins as growth factors regulating stem development may be affected, resulting in changes in stem growth and development. Accumulation of both proline and chlorophyll may be regulated by PagERF transcription factors, allowing control of H_2_O_2_ content and stomatal conductance to resist drought stress in leaves [57]. Therefore, PagERF transcription factors play an important role in transcriptional regulation in the molecular network of *P. alba × P. glandulosa* in response to drought stress.

**Figure 10:**
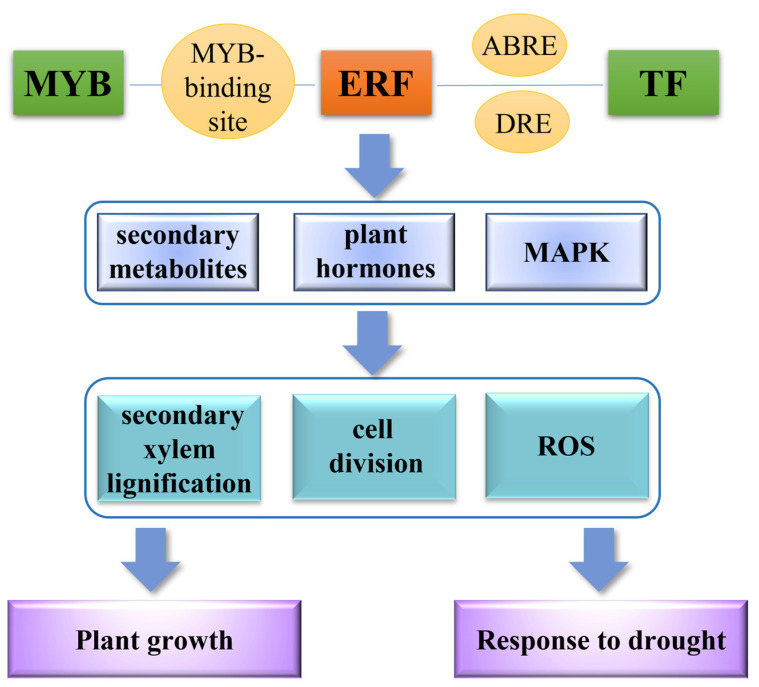
Model of ERF function in plant growth and response to drought.

We conclude that PagERF transcription factors play a key regulatory role in the growth and development of *P. alba × P. glandulosa* and the molecular network in response to drought stress (Figure 10). Taken together, these investigations benefit the selection of potentially improved PagERF transcription factors in the regulation of drought responses in *P. alba × P. glandulosa* and help to improve our understanding of the biological function of the ERF transcription factor family. However, in view of *P. alba × P. glandulosa* possessing two subgenomes, the interaction between PagERF transcription factors and their regulatory network relationships with other transcription factors or functional genes need to be further explored in the future.

## 4. Materials and Methods

### 4.1. Plant Materials and Treatments

Clonal wild-type *P. alba × P. glandulosa* (84K poplar) seedling tops with axillary buds and one single leaf were placed into ½-strength Murashige and Skoog (MS) medium supplemented with 0.1 mg/L indole-3-butyric acid (IBA) and 0.01 mg/L 1-naphthaleneacetic acid (NAA) and grown under long-day conditions (16-h light/8-h dark) at 23–25 °C for 20 days. For simulated drought stress, 20-day-old sterile seedlings were placed into 1/2 MS medium containing 6% (*w*/*v*) PEG6000 without exogenous hormones. Axillary buds, young leaves, functional leaves, stems, and roots were collected at 0, 3, 6, 12, 24, 48, 96, and 128 h from the onset of treatment and stored in liquid nitrogen for RNA extraction. At least three biological replicates were performed for each group.

### 4.2. Identification of PagERF Transcription Factors

The genomes of *P. alba × P. glandulosa* were referenced as published [31], and *A. thaliana* AP2/ERF full-length protein sequences were obtained from The Arabidopsis Information Resource database (TAIR; https://www.arabidopsis.org/) (accessed on 15 October 2022). AP2/ERF proteins in *P. alba × P. glandulosa* were identified using BLAST (e-value, 1 × e^−5^) in TBtools software (version 1.098769) and those with sequence identity (percentage identity) greater than 80% were retained (Supplementary Table S11). All proteins retrieved using BLAST were then further analyzed using Batch NCBI CD-Search Tools (https://www.ncbi.nlm.nih.gov/Structure/bwrpsb/bwrpsb.cgi) (accessed on 15 October 2022) to identify conserved AP2 domains. Visualization was performed using Visualize NCBI CDD Domain Pattern in the TBtools software. Number of amino acids, molecular weight, theoretical pI, instability index, aliphatic index, and grand average of hydropathicity were analyzed using Protein Parameter Calc (ProtParam-based) in the TBtools software, and subcellular localization of PagERF transcription factors was analyzed using Plant-mPLoc (http://www.csbio.sjtu.edu.cn/bioinf/plant-multi/) (version 2.0) (accessed on 15 October 2022).

### 4.3. Chromosomal Localization and Evolutionary Analysis of PagERF Transcription Factors

Visual maps of 19 chromosomes from subgenomes A and B of *P. alba × P. glandulosa* based on General Feature Format (GFF) information in the TBtools software were analyzed, respectively, and replicated, duplicated, and orthologous pairs of *PagERF* genes were analyzed using MCScanX and Advanced Circos in TBtools software. The location of *PagERF* genes on chromosomes was analyzed using Gene Location Visualize from GTF/GFF in TBtools software.

### 4.4. Motif Analysis of PagERF Transcription Factors and Phylogenetic Tree Reconstruction

The conserved motifs of PagERF transcription factors were identified using MEME (http://meme-suite.org/tools/meme) (version 5.5.0) (accessed on 15 October 2022); 10 motifs were analyzed, and the acquiescent minimum and maximum width of motifs was set from 6 to 50. A phylogenetic tree was reconstructed using PagERF protein sequences identified by ClustalW algorithm in MEGA (version 7.0.26) software with the method of neighbor-joining algorithm and the parameters of pairwise deletion and 1000 replicates for bootstrap analysis. Interactive Tree of Life (iTOL) (https://itol.embl.de/) (version 6.6) (accessed on 15 October 2022) was used to further process the phylogenetic tree. Gene Structure View (Advanced) in TBtools software was used for visualization. The function of motifs was analyzed by InterPro (https://www.ebi.ac.uk/interpro/) (accessed on 15 October 2022).

### 4.5. Analysis of Cis-Acting Elements in PagERF Promoters

Regions 2000 bp upstream of *PagERF* coding sequences were selected as promoter sequences using the Gtf/Gff3 Sequences Extractor in TBtools software, and cis-acting elements of *PagERF* promoters were analyzed using PlantCARE (http://bioinformatics.psb.ugent.be/webtools/plantcare/html/) (version 1) (accessed on 15 October 2022). Simple BioSequence Viewer in TBtools software was used for visualization.

### 4.6. RNA Extraction and RT-qPCR

Total RNA from different plant material (axillary buds, young leaves, functional leaves, stems, cambium, xylem and roots) was extracted using a Qiagen RNeasy Plant Mini Kit (QIAGEN). DNA was digested using an RNase-Free DNase Set (QIAGEN) during the process of RNA extraction. Genes were selected for RT-qPCR analysis with specific primers (Supplementary Table S12). RT-qPCR primers were designed by Integrated DNA Technologies (IDT) database tools (https://sg.idtdna.com/Scitools/Applications/RealTimePCR/) (accessed on 15 October 2022), and primer specificities were tested by executing a Blastn search against local *P. alba × P. glandulosa* genome data. A total of 200 ng RNA was used for synthesizing cDNA using a PrimeScript RT reagent kit (TaKaRa); this cDNA was used for RT-qPCR analysis. Real-time PCR was performed on an Agilent M × 3000P Real-Time PCR System using a TB Green Premix ExTaq II (Tli RNaseH Plus) kit (TaKaRa). Three biological replicates and three technical replicates of each reaction were performed. The RT-qPCR procedure was as follows: pre-denaturation at 95 °C for 10 min and 40 cycles of 95 °C for 30 s, 60 °C for 1 min. Experimental data were processed using the 2^−ΔΔCT^ method [58].

### 4.7. RNA Sequencing

Total RNA of multiple samples were grouped together in three biological replicates: axillary buds (WT_axillary bud_1, WT_axillary bud_2, WT_axillary bud_3), young leaves (WT_ young leaf_1, WT_ young leaf_2, WT_ young leaf_3), functional leaves (WT_ functional leaf_1, WT_ functional leaf_2, WT_ functional leaf_3), cambium (WT_cambium_1, WT_ cambium_2, WT_ cambium_3), xylem (WT_xylem_1, WT_ xylem _2, WT_ xylem _3) and roots (WT_root_1, WT_ root _2, WT_ root _3). This project uses Huada’s self-developed filtering software SOAPnuke (v1.4.0) for filtering. The specific steps are as follows: (I) Remove the reads containing the connector (connector contamination); (II) Remove reads with unknown base N content greater than 5%; (III) Remove low-quality reads (we define low-quality reads as reads with a base mass value below 15 accounting for more than 20% of the total base number of the reads). After obtaining clean reads, HISAT (v2.1.0) was used to align clean reads to the reference genome sequence (Supplementary Table S13).

## 5. Conclusions

We identified members of the PagERF family in *P. alba × P. glandulosa* and analyzed the expression patterns of *PagERF* genes in axillary buds, young leaves, functional leaves, cambium, xylem, and roots, as well as expression patterns in response to drought stress. We characterized the conserved domains, physicochemical properties, gene structure, evolutionary relationships, and gene replication relationships on chromosomes of 209 PagERF transcription factors. Moreover, we used transcriptome data and quantitative RT-qPCR to verify the expression patterns of *PagERF* genes in different tissues of *P. alba × P. glandulosa*, showing that most *PagERF* genes are expressed strongly in roots. We observed the expression patterns of nine *PagERF* genes in different tissues in response to drought simulated using 6% PEG6000. This revealed that *PagERF* genes display different degrees of response to drought stress in various tissues. These results indicate that PagERF transcription factors play an important regulatory role in the molecular network of *P. alba × P. glandulosa* growth, development, and drought response.

## Data Availability

The RNA-seq raw sequencing data have been deposited in the National Center for Biotechnology Information Sequence Read Archive under accession number PRJNA916663.

## Supplementary Materials

The following supporting information can be downloaded at: https://www.mdpi.com/article/10.3390/ijms24043697/s1.

## References

[1] Ambawat S., Sharma P., Yadav N.R., Yadav R.C. (2013). MYB transcription factor genes as regulators for plant responses: An overview. Physiol. Mol. Biol. Plants.

[2] Wang H., Wang H., Shao H., Tang X. (2016). Recent Advances in Utilizing Transcription Factors to Improve Plant Abiotic Stress Tolerance by Transgenic Technology. Front Plant Sci..

[3] Li X., Gao B., Zhang D., Liang Y., Liu X., Zhao J., Zhang J., Wood A.J. (2018). Identification, Classification, and Functional Analysis of AP2/ERF Family Genes in the Desert Moss Bryum argenteum. Int. J. Mol. Sci..

[4] Liu M., Sun W., Ma Z., Zheng T., Huang L., Wu Q., Zhao G., Tang Z., Bu T., Li C. (2019). Genome-wide investigation of the AP2/ERF gene family in tartary buckwheat (*Fagopyum Tataricum*). BMC Plant Biol..

[5] Du D., Hao R., Cheng T., Pan H., Yang W., Wang J., Zhang Q. (2012). Genome-Wide Analysis of the AP2/ERF Gene Family in Prunus mume. Plant Mol. Biol. Report..

[6] Wu H., Lv H., Li L., Liu J., Mu S., Li X., Gao J. (2015). Genome-Wide Analysis of the AP2/ERF Transcription Factors Family and the Expression Patterns of DREB Genes in Moso Bamboo (*Phyllostachys edulis*). PLoS ONE.

[7] Yu Y., Yu M., Zhang S., Song T., Zhang M., Zhou H., Wang Y., Xiang J., Zhang X. (2022). Transcriptomic Identification of Wheat AP2/ERF Transcription Factors and Functional Characterization of TaERF-6-3A in Response to Drought and Salinity Stresses. Int. J. Mol. Sci..

[8] Wang P., Cheng T., Lu M., Liu G., Li M., Shi J., Lu Y., Laux T., Chen J. (2016). Expansion and functional divergence of AP2 group genes in spermatophytes determined by molecular evolution and Arabidopsis mutant analysis. Front. Plant Sci..

[9] Jiang W., Zhang X., Song X., Yang J., Pang Y. (2020). Genome-Wide Identification and Characterization of APETALA2/Ethylene-Responsive Element Binding Factor Superfamily Genes in Soybean Seed Development. Front Plant Sci..

[10] Xie Z., Yang C., Liu S., Li M., Gu L., Peng X., Zhang Z. (2022). Identification of AP2/ERF transcription factors in Tetrastigma hemsleyanum revealed the specific roles of ERF46 under cold stress. Front. Plant Sci..

[11] Wang S., Yao W., Zhou B., Jiang T. (2016). Structure analysis and expression pattern of the ERF transcription factor family in poplar. Acta Physiol. Plant..

[12] Li Q., Zhang L., Chen P., Wu C., Zhang H., Yuan J., Zhou J., Li X. (2022). Genome-Wide Identification of APETALA2/ETHYLENE RESPONSIVE FACTOR Transcription Factors in Cucurbita moschata and Their Involvement in Ethylene Response. Front Plant Sci..

[13] Zhao M., Haxim Y., Liang Y., Qiao S., Gao B., Zhang D., Li X. (2022). Genome-wide investigation of AP2/ERF gene family in the desert legume Eremosparton songoricum: Identification, classification, evolution, and expression profiling under drought stress. Front Plant Sci..

[14] Zhang L., Chen L., Pang S., Zheng Q., Quan S., Liu Y., Xu T., Liu Y., Qi M. (2022). Function Analysis of the ERF and DREB Subfamilies in Tomato Fruit Development and Ripening. Front Plant Sci..

[15] Guo B., Wei Y., Xu R., Lin S., Luan H., Lv C., Zhang X., Song X., Xu R. (2016). Genome-Wide Analysis of APETALA2/Ethylene-Responsive Factor (AP2/ERF) Gene Family in Barley (*Hordeum vulgare* L.). PLoS ONE.

[16] Zhang J., Liao J., Ling Q., Xi Y., Qian Y. (2022). Genome-wide identification and expression profiling analysis of maize AP2/ERF superfamily genes reveal essential roles in abiotic stress tolerance. BMC Genom..

[17] Karanja B.K., Xu L., Wang Y., Tang M., M’Mbone Muleke E., Dong J., Liu L. (2019). Genome-wide characterization of the AP2/ERF gene family in radish (*Raphanus sativus* L.): Unveiling evolution and patterns in response to abiotic stresses. Gene.

[18] Nie J., Wen C., Xi L., Lv S., Zhao Q., Kou Y., Ma N., Zhao L., Zhou X. (2018). The AP2/ERF transcription factor CmERF053 of chrysanthemum positively regulates shoot branching, lateral root, and drought tolerance. Plant Cell Rep..

[19] Javed T., Shabbir R., Ali A., Afzal I., Zaheer U., Gao S.-J. (2020). Transcription Factors in Plant Stress Responses: Challenges and Potential for Sugarcane Improvement. Plants.

[20] Dubois M., Van den Broeck L., Inze D. (2018). The Pivotal Role of Ethylene in Plant Growth. Trends Plant Sci..

[21] Xie Z., Nolan T., Jiang H., Tang B., Zhang M., Li Z., Yin Y. (2019). The AP2/ERF Transcription Factor TINY Modulates Brassinosteroid-Regulated Plant Growth and Drought Responses in Arabidopsis. Plant Cell.

[22] Erpen L., Devi H.S., Grosser J.W., Dutt M. (2017). Potential use of the DREB/ERF, MYB, NAC and WRKY transcription factors to improve abiotic and biotic stress in transgenic plants. Plant Cell Tissue Organ Cult. (PCTOC).

[23] Golldack D., Li C., Mohan H., Probst N. (2014). Tolerance to drought and salt stress in plants: Unraveling the signaling networks. Front Plant Sci..

[24] Jung H., Chung P.J., Park S.H., Redillas M., Kim Y.S., Suh J.W., Kim J.K. (2017). Overexpression of OsERF48 causes regulation of OsCML16, a calmodulin-like protein gene that enhances root growth and drought tolerance. Plant Biotechnol. J..

[25] Jan R., Asaf S., Numan M., Kim K.M. (2021). Plant Secondary Metabolite Biosynthesis and Transcriptional Regulation in Response to Biotic and Abiotic Stress Conditions. Agronomy.

[26] An J.P., Zhang X.W., Bi S.Q., You C.X., Wang X.F., Hao Y.J. (2020). The ERF transcription factor MdERF38 promotes drought stress-induced anthocyanin biosynthesis in apple. Plant J..

[27] Zhao Y., Guo Q., Cao S., Tian Y., Han K., Sun Y., Li J., Yang Q., Ji Q., Sederoff R. (2022). Genome-wide identification of the AlkB homologs gene family, PagALKBH9B and PagALKBH10B regulated salt stress response in Populus. Front. Plant Sci..

[28] Yan H., Zhang X., Li X., Wang X., Li H., Zhao Q., Yin P., Guo R., Pei X., Hu X. (2022). Integrated Transcriptome and Metabolome Analyses Reveal the Anthocyanin Biosynthesis Pathway in AmRosea1 Overexpression 84K Poplar. Front Bioeng Biotechnol..

[29] Ren Y., Zhang S., Xu T., Kang X. (2022). Morphological, Transcriptome, and Hormone Analysis of Dwarfism in Tetraploids of Populus alba x P. glandulosa. Int. J. Mol. Sci..

[30] Xia Y., Du K., Ling A., Wu W., Li J., Kang X. (2022). Overexpression of PagSTOMAGEN, a Positive Regulator of Stomatal Density, Promotes Vegetative Growth in Poplar. Int. J. Mol. Sci..

[31] Qiu D., Bai S., Ma J., Zhang L., Shao F., Zhang K., Yang Y., Sun T., Huang J., Zhou Y. (2019). The genome of Populus alba × Populus tremula var. glandulosa clone 84K. DNA Res..

[32] Huang X., Chen S., Peng X., Bae E.-K., Dai X., Liu G., Qu G., Ko J.-H., Lee H., Chen S. (2020). An improved draft genome sequence of hybrid Populus alba × Populus glandulosa. J. For. Res..

[33] Liao W., Li Y., Yang Y., Wang G., Peng M. (2016). Exposure to various abscission-promoting treatments suggests substantial ERF subfamily transcription factors involvement in the regulation of cassava leaf abscission. BMC Genom..

[34] He S., Hao X., He S., Hao X., Zhang P., Chen X. (2021). Genome-wide identification, phylogeny and expression analysis of AP2/ERF transcription factors family in sweet potato. BMC Genom..

[35] Vahala J., Felten J., Love J., Gorzsas A., Gerber L., Lamminmaki A., Kangasjarvi J., Sundberg B. (2013). A genome-wide screen for ethylene-induced ethylene response factors (ERFs) in hybrid aspen stem identifies ERF genes that modify stem growth and wood properties. New Phytol..

[36] Fan W., Hai M., Guo Y., Ding Z., Tie W., Ding X., Yan Y., Wei Y., Liu Y., Wu C. (2016). The ERF transcription factor family in cassava: Genome-wide characterization and expression analyses against drought stress. Sci. Rep..

[37] Zhuang J., Cai B., Peng R.H., Zhu B., Jin X.F., Xue Y., Gao F., Fu X.Y., Tian Y.S., Zhao W. (2008). Genome-wide analysis of the AP2/ERF gene family in Populus trichocarpa. Biochem Biophys Res. Commun..

[38] Sun X., Wu W., Yang Y., Wilson I., Shao F., Qiu D. (2022). Genome-Wide Identification of m(6)A Writers, Erasers and Readers in Poplar 84K. Genes.

[39] Ji A.J., Luo H.M., Xu Z.C., Zhang X., Zhu Y.J., Liao B.S., Yao H., Song J.Y., Chen S.L. (2016). Genome-Wide Identification of the AP2/ERF Gene Family Involved in Active Constituent Biosynthesis in *Salvia miltiorrhiza*. Plant Genome.

[40] Ya-Ting H., Zhi-Chao X., Ya T., Ran-Ran G., Ai-Jia J., Xiang-Dong P., Yu W., Xia L., Jing-Yuan S. (2020). Genome-wide identification and analysis of AP2/ERF transcription factors related to camptothecin biosynthesis in Camptotheca acuminata. Chin. J. Nat. Med..

[41] Liu M., Diretto G., Pirrello J., Roustan J.P., Li Z., Giuliano G., Regad F., Bouzayen M. (2014). The chimeric repressor version of an Ethylene Response Factor (ERF) family member, Sl-ERF.B3, shows contrasting effects on tomato fruit ripening. New Phytol..

[42] Xu S., Yao S., Huang R., Tan Y., Huang D. (2020). Transcriptome-wide analysis of the AP2/ERF transcription factor gene family involved in the regulation of gypenoside biosynthesis in Gynostemma pentaphyllum. Plant Physiol. Biochem.

[43] Duan C., Argout X., Gébelin V., Summo M., Dufayard J.-F., Leclercq J., Piyatrakul P., Pirrello J., Rio M., Champion A. (2013). Identification of the Hevea brasiliensisAP2/ERF superfamily by RNA sequencing. BMC Genom..

[44] Dubois M., Skirycz A., Claeys H., Maleux K., Dhondt S., De Bodt S., Vanden Bossche R., De Milde L., Yoshizumi T., Matsui M. (2013). Ethylene Response Factor6 acts as a central regulator of leaf growth under water-limiting conditions in Arabidopsis. Plant Physiol..

[45] Rupp H.M. (2013). Effects of Ethylene on Secondary Xylem Formation in Arabidopsis Thaliana.

[46] Lee D.K., Jung H., Jang G., Jeong J.S., Kim Y.S., Ha S.H., Do Choi Y., Kim J.K. (2016). Overexpression of the OsERF71 Transcription Factor Alters Rice Root Structure and Drought Resistance. Plant Physiol..

[47] Li M.Y., Xu Z.S., Huang Y., Tian C., Wang F., Xiong A.S. (2015). Genome-wide analysis of AP2/ERF transcription factors in carrot (*Daucus carota* L.) reveals evolution and expression profiles under abiotic stress. Mol. Genet Genom..

[48] Liu C., Zhang T. (2017). Expansion and stress responses of the AP2/EREBP superfamily in cotton. BMC Genom..

[49] Hu F., Zhang Y., Guo J. (2022). Identification and Characterization of AP2/ERF Transcription Factors in Yellow Horn. Int. J. Mol. Sci..

[50] Chen Y., Yang J., Wang Z., Zhang H., Mao X., Li C. (2013). Gene structures, classification, and expression models of the DREB transcription factor subfamily in Populus trichocarpa. Sci. World J..

[51] Zhao M., Li Y., Zhang X., You X., Yu H., Guo R., Zhao X. (2022). Genome-Wide Identification of AP2/ERF Superfamily Genes in Juglans mandshurica and Expression Analysis under Cold Stress. Int. J. Mol. Sci..

[52] Wu Y., Zhang L., Nie L., Zheng Y., Zhu S., Hou J., Li R., Chen G., Tang X., Wang C. (2022). Genome-wide analysis of the DREB family genes and functional identification of the involvement of BrDREB2B in abiotic stress in wucai (*Brassica campestris* L.). BMC Genom..

[53] Zhou L., Yarra R. (2021). Genome-Wide Identification and Characterization of AP2/ERF Transcription Factor Family Genes in Oil Palm under Abiotic Stress Conditions. Int. J. Mol. Sci..

[54] Xie Z., Nolan T.M., Jiang H., Yin Y. (2019). AP2/ERF Transcription Factor Regulatory Networks in Hormone and Abiotic Stress Responses in Arabidopsis. Front Plant Sci..

[55] Kazan K. (2015). Diverse roles of jasmonates and ethylene in abiotic stress tolerance. Trends Plant Sci..

[56] Love J., Björklund S., Vahala J., Hertzberg M., Kangasjärvi J., Sundberg B. (2009). Ethylene is an endogenous stimulator of cell division in the cambial meristem of Populus. Proc. Natl. Acad. Sci. USA.

[57] Rong W., Qi L., Wang A., Ye X., Du L., Liang H., Xin Z., Zhang Z. (2014). The ERF transcription factor TaERF3 promotes tolerance to salt and drought stresses in wheat. Plant Biotechnol. J..

[58] Livak K.J., Schmittgen T.D. (2001). Analysis of relative gene expression data using real-time quantitative PCR and the 2(-Delta Delta C(T)) Method. Methods.

