# Supplementary material for: Genome-Wide Identification of the ERF Transcription Factor Family for Structure Analysis, Expression Pattern, and Response to Drought Stress in Populus alba × Populus glandulosa
Source: Int J Mol Sci. 2023 Feb 12;24(4):3697. doi: 10.3390/ijms24043697 (PMC9967527; doi:10.3390/ijms24043697)
Supplement: Supplementary file 1 [file ijms-24-03697-s001.zip › ijms-2179743-supplementary.pdf]

Supplementary Material

1 Supplementary Figures

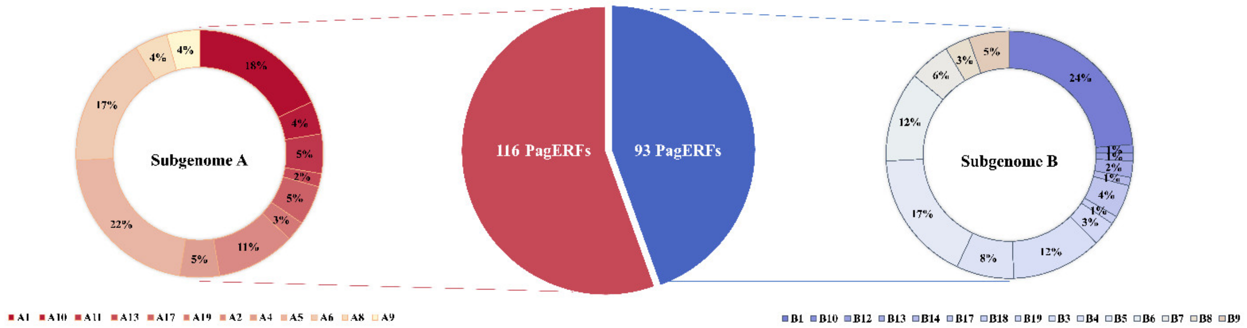

Supplementary Figure S1. The distribution of *PagERFs* on each chromosome in subgenome A and B. The red doughnut chart represents distribution ratio of *PagERFs* on chromosomes in subgenome A. The blue doughnut chart represents distribution ratio of *PagERFs* on chromosomes in subgenome B. The pie chart represents total number of *PagERFs* in subgenome A and B. The figure legends of A1 to A19 represent chromosome 1 to 19 for subgenome A, and the figure legends of B1 to B19 represent chromosome 1 to 19 for subgenome B.

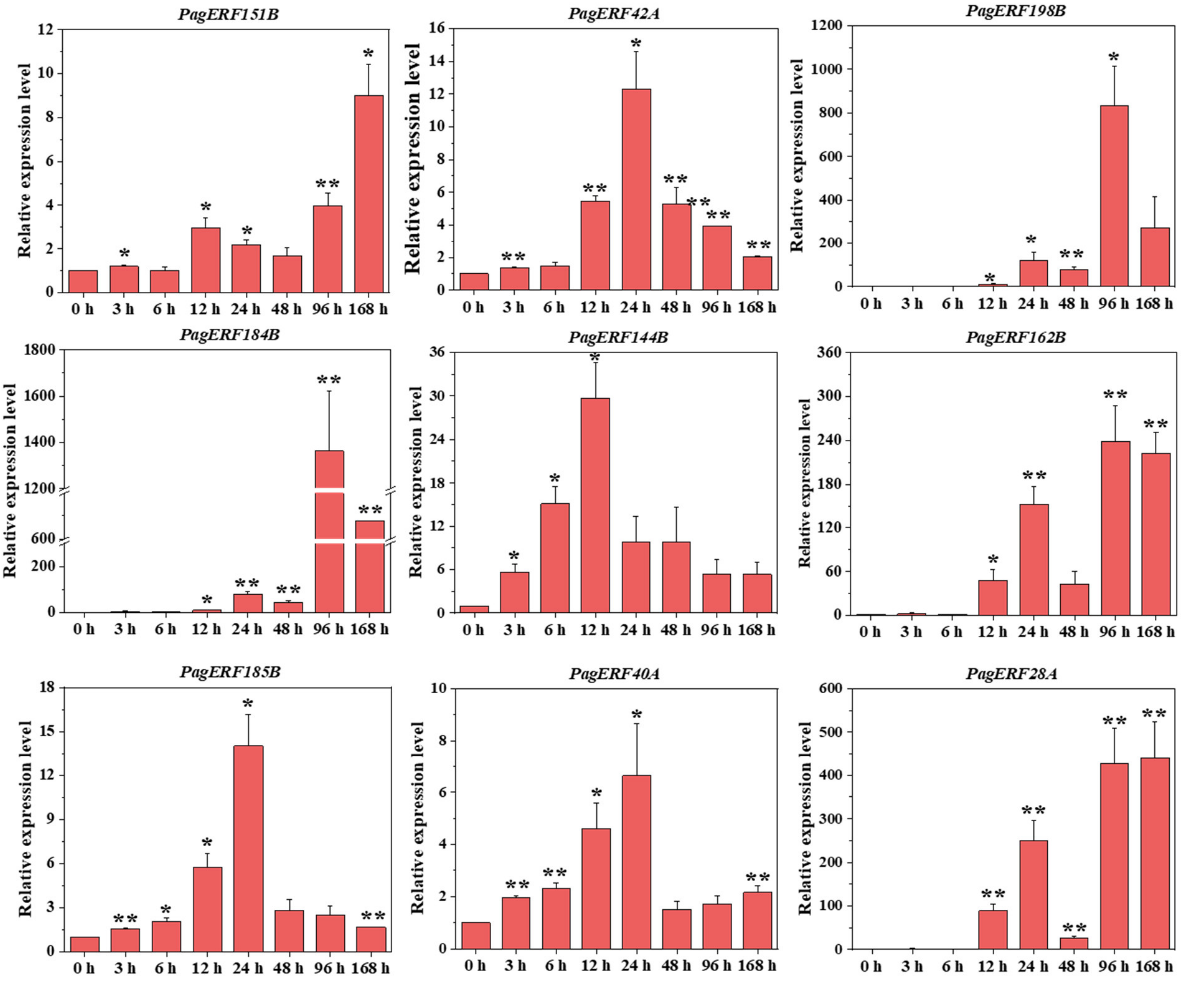

Supplementary Figure S2. Quantitative validation of expression patterns for nine *PagERF* genes in axillary bud under drought stress at eight time points. The X-axis represents the samples under drought stress at eight time points. The Y-axis on the left indicates the relative gene expression levels analyzed by qRT-PCR. The bars indicate mean ± SE (n = 3) from three independent trials.

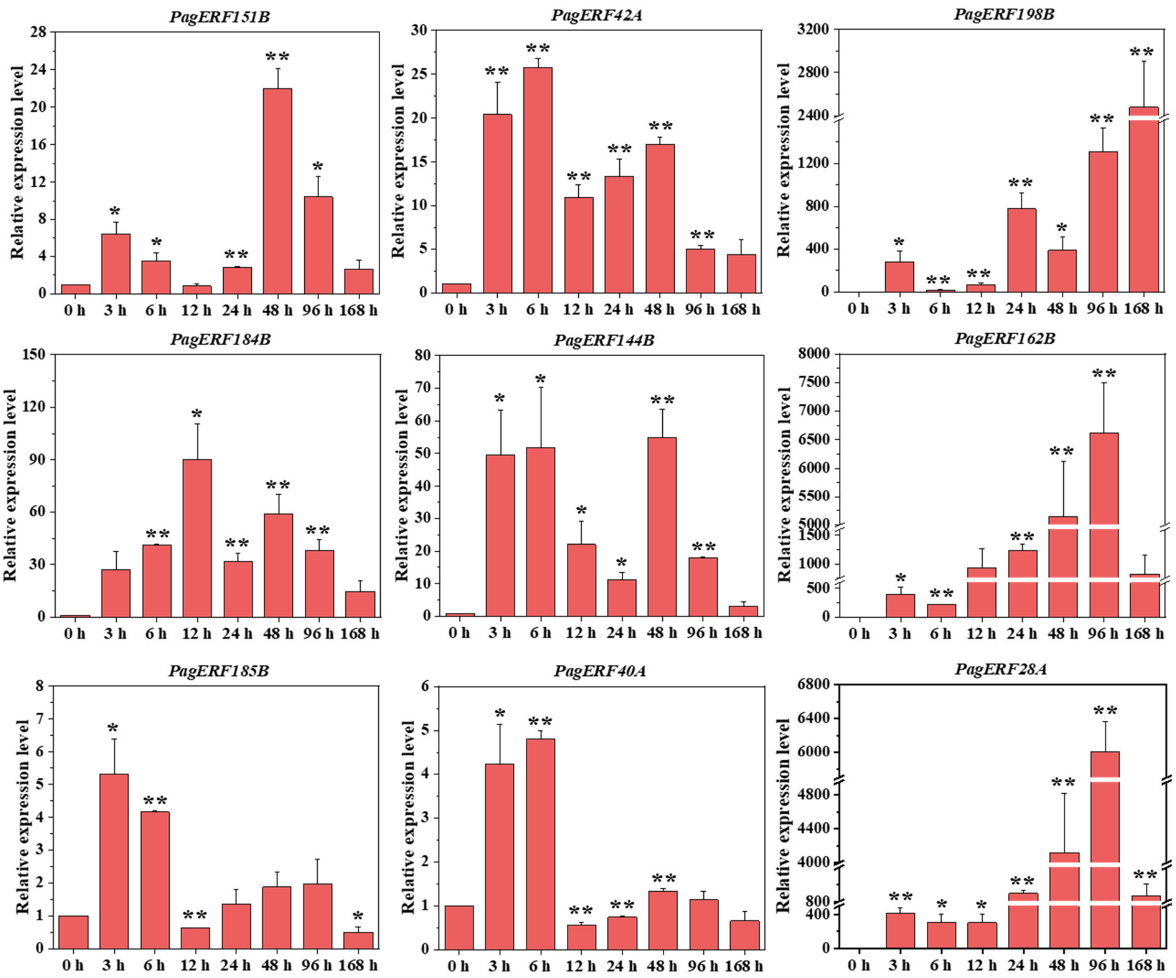

Supplementary Figure S3. Quantitative validation of expression patterns for nine *PagERF* genes in young leaf under drought stress at eight time points. The X-axis represents the samples under drought stress at eight time points. The Y-axis on the left indicates the relative gene expression levels analyzed by qRT-PCR. The bars indicate mean ± SE (n = 3) from three independent trials.

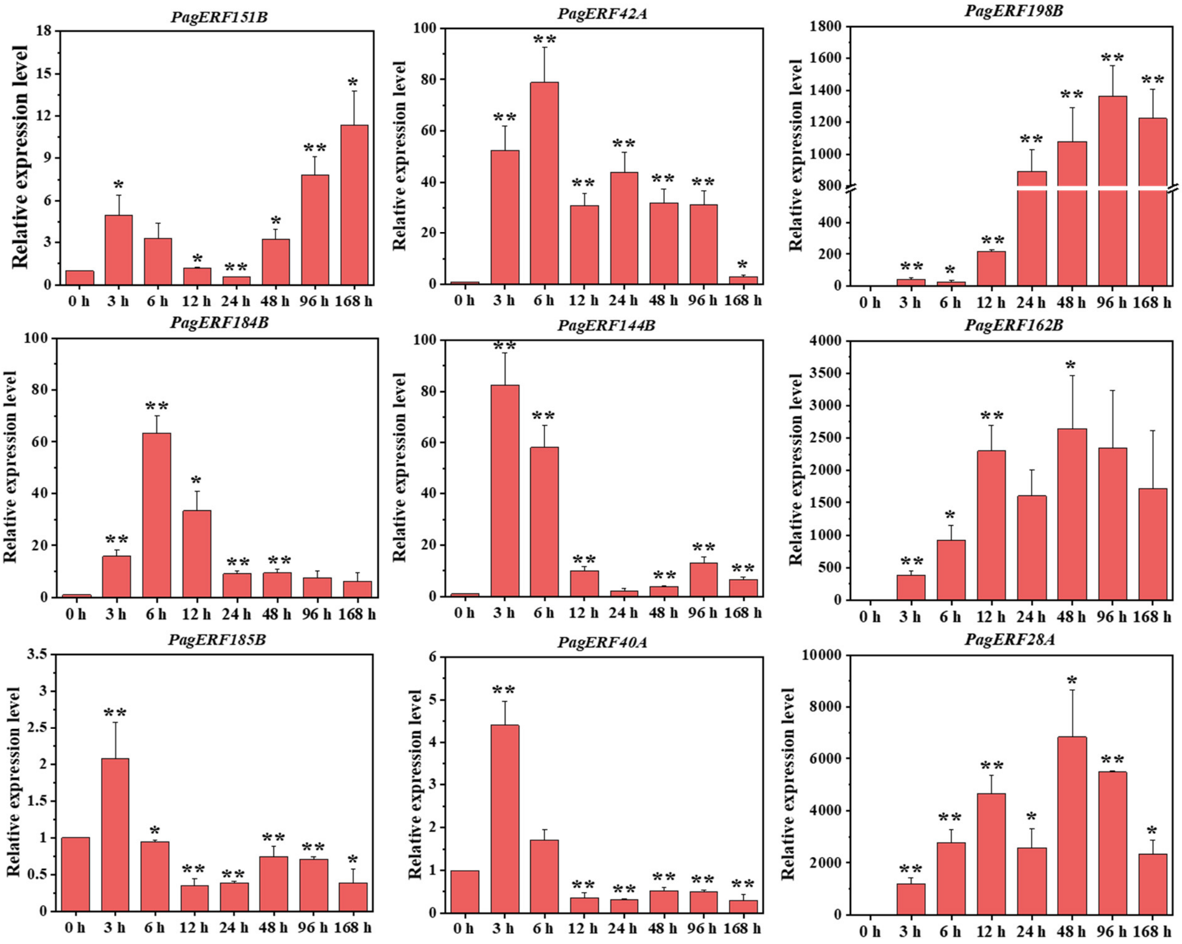

Supplementary Figure S4. Quantitative validation of expression patterns for nine *PagERF* genes in functional leaf under drought stress at eight time points. The X-axis represents the samples under drought stress at eight time points. The Y-axis on the left indicates the relative gene expression levels analyzed by qRT-PCR. The bars indicate mean ± SE (n = 3) from three independent trials.

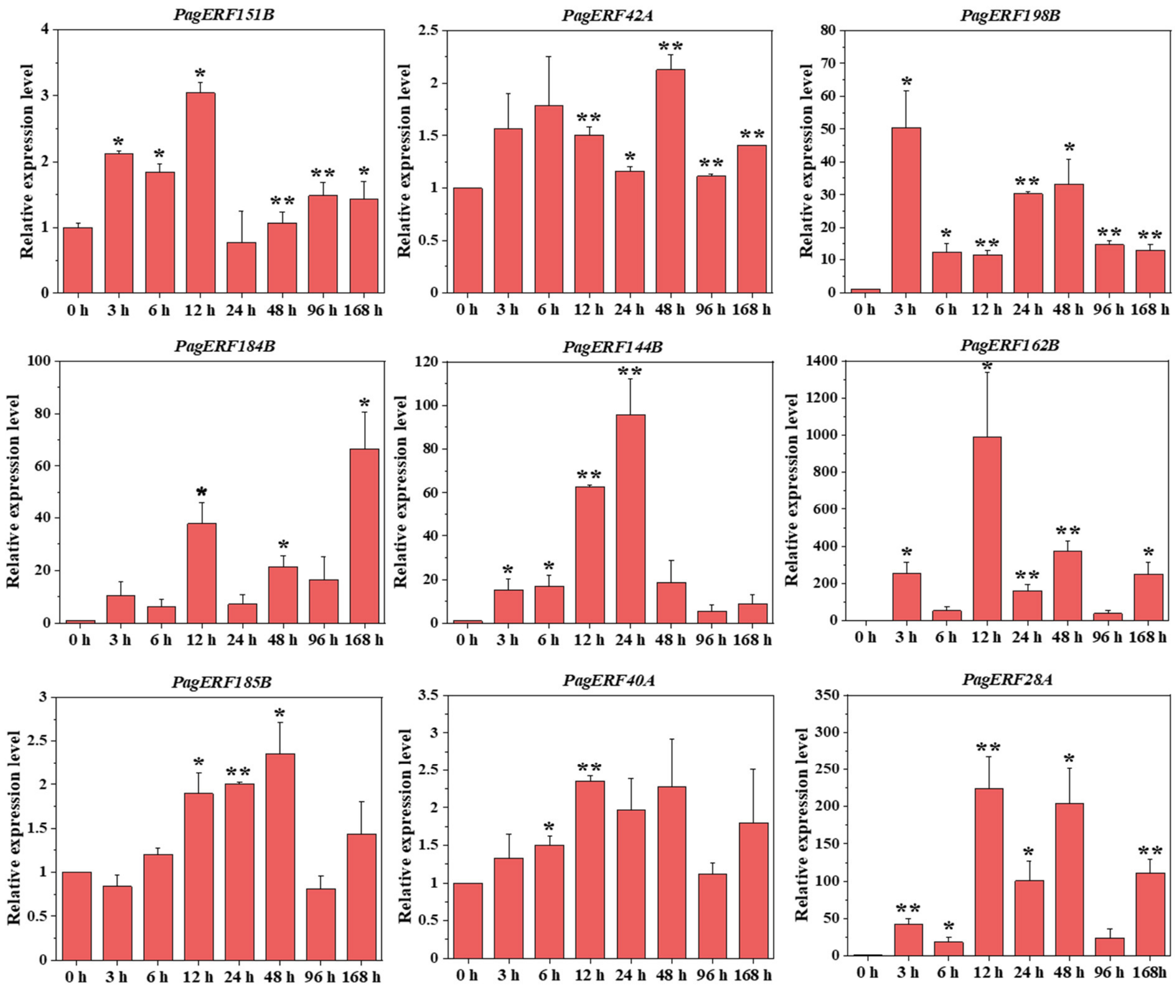

Supplementary Figure S5. Quantitative validation of expression patterns for nine *PagERF* genes in stem under drought stress at eight time points. The X-axis represents the samples under drought stress at eight time points. The Y-axis on the left indicates the relative gene expression levels analyzed by qRT-PCR. The bars indicate mean ± SE (n = 3) from three independent trials.
